# Spatio-temporal occurrence of small carnivores in Bagmati-Ratu Complex, Nepal

**DOI:** 10.1371/journal.pone.0362681

**Published:** 2026-11-13

**Authors:** Bashu Dev Baral, Surya Devkota, Sandeep Regmi, Shivish Bhandari, Bishnu Prasad Bhattarai, Hem Bahadur Katuwal, Jerrold L. Belant, Hari Prasad Sharma

**Affiliations:** 1 Central Department of Zoology, Institute of Science and Technology, Tribhuvan University, Kirtipur, Kathmandu, Nepal; 2 Nepal Zoological Society, Kirtipur, Kathmandu, Nepal; 3 Southeast Asia Biodiversity Research Institute, Chinese Academy of Sciences and Center for Integrative Conservation, Xishuangbanna Tropical Botanical Garden, Chinese Academy of Sciences, Mengla, Yunnan, China; 4 The Himalayan Conservancy, Kirtipur, Nepal; 5 Morgan State University, Baltimore, Maryland, United States of America; 6 Wild Harvest Institute, Michigan State University, East Lansing, Michigan, United States of America; Xinjiang Medical University Affiliated First Hospital, CHINA

## Abstract

Small carnivores are generally more adaptable to human-dominated landscapes compared with large carnivores, but have received comparatively limited attention. We evaluated the single-season multi-species occupancy and temporal overlap of five small carnivores in Bagmati-Ratu Complex (BRC) of Madhesh Province, Nepal using camera-trap surveys during January to February 2023. Camera-trap data were used to evaluate single-season multi-species occupancy using Markov Chain Monte Carlo simulations to understand the impacts of anthropogenic and environmental variables on community-level occupancy of small carnivores. We also calculated pairwise activity overlap coefficients (Dhat1) to examine temporal interactions among species. We observed the highest detection probability for jungle cat (*Felis chaus*; p = 0.315), whereas the lowest detection probability was for leopard cat (*Prionailurus bengalensis*; p = 0.245). Occupancy was highest for common palm civet (0.698), whereas lowest for large Indian civet (*Viverra zibetha*; 0.651). The community-level occupancy was negatively associated with area of farmland, whereas settlement area was positively associated at species-level only. All species exhibited strong nocturnal activity patterns, except the jungle cat, which showed some diurnal activity. We found high temporal overlap between the common palm civet and small Indian civet, consistent with their nocturnal behavior (Dhat1 = 0.77). All small carnivores exhibited similar activity patterns, with the reduced activity during the daytime and increased activity from dusk through dawn, indicating strongly nocturnal behavior and suggesting temporal avoidance of humans. These baseline data provide valuable information for understanding the coexistence of humans and small carnivores and can inform future conservation strategies.

## 1. Introduction

Small carnivores are integral components of ecosystems, contributing to ecosystem functioning and the maintenance of ecological processes [1,2]. For example, they maintain ecosystem structure through activities like seed dispersal [3,4] and affect nutrient (nitrogen and phosphorus) levels in soils which ultimately leads to modification of plant communities [5]. Small carnivores also help humans by maintaining populations of rodent pests in farmlands and settlements [6] and can reduce the potential of disease transmission to humans [7]. Further, predation of small carnivores by top predators facilitates the transfer of energy between the carnivores [2,8]. Despite their importance, small carnivores experience multiple anthropogenic threats including habitat degradation and illegal harvests [9–12] and vehicular accidents [13,14]. Considering the ecological importance of small carnivores and the ongoing threats they face, it is important to understand factors that shape their occurrence and resource use.

The occurrence and resource use of wildlife are influenced by habitat availability, food and water resources, and levels of disturbances [15–17]. Previous studies have shown that small carnivores exhibit species-specific responses to environmental and anthropogenic factors, with the direction and magnitude of these relationships varying among species and landscapes. For example, the occupancy of leopard cats and jungle cats in Dhorpatan Hunting Reserve, Nepal, increased with canopy cover and livestock detections but decreased with increasing distance from human settlements [16]. Similarly, the occurrence of leopard cats was positively associated with higher anthropogenic influence and the presence of other cat species in Langtang National Park, Nepal [18]. In contrast, the occupancy of jungle cat decreased with increasing canopy cover in the Parsa-Koshi Complex, Nepal [19], and was negatively affected by forest cover and distance to water sources in Tadoba-Andhari Tiger Reserve, India [20]. Likewise, the occupancy of large Indian civet in Bayuelin Nature Reserve, China, was positively associated with human activities and negatively associated with distance from rivers and roads [21]. Despite numerous studies on environmental and anthropogenic factors influencing small carnivore occurrence, information on their occurrence and responses to such factors in human-dominated landscapes remains limited, particularly outside protected areas, even though small carnivores are more threatened than larger carnivores [12].

To understand species occurrence and its associated factors, robust analytical approaches, such as occupancy modelling are required, as they account for imperfect detection and provide more reliable inferences than traditional presence–absence surveys [16,22,23]. Although most occupancy studies have focused on single species [24–28], multi-species occupancy models have emerged as powerful tools for studying communities as well as species of conservation concern while quantifying the effects of environmental factors and interspecific interactions that shape species occurrence [16,29–31].

The Bagmati-Ratu Complex (BRC) is a human-dominated landscape that lies entirely outside protected areas (PA) in the lowland Tarai of Nepal, where small carnivores and humans share common resources. The community and national forests within the BRC provide habitat for small carnivores despite ongoing anthropogenic pressure, including expansion of farmland and human settlements [32], as well as high livestock grazing, tree cutting and forest fire [33]. Furthermore, small carnivores frequently prey on poultry near human settlements, thereby potentially exacerbating human–carnivore conflict [8,34]. Given all these in considerations, understanding small carnivore occurrence in the BRC is important for their conservation and coexistence.

Furthermore, species with similar morphology and ecology living within the same habitat develop coexistence strategies, including dietary, temporal or spatial partitioning [19,35,36].To date, few studies have attempted to understand the temporal activity patterns of small carnivore communities [37,38] as most studies either focus on only one or two species [19,39–41] or evaluate them in relation to predator presence [41–43], limiting our understanding of these patterns at the community level.

Given the limited understanding of small carnivore ecology in human-dominated landscapes outside protected areas, this study aimed to (1) quantify single-season, multi-species occupancy of five small carnivores across the BRC using camera-trap data; (2) assess the effects of environmental and anthropogenic factors on species and community-level occupancy; and (3) investigate diel activity patterns and temporal overlap among jungle cat (*Felis chaus*), leopard cat (*Prionailurus bengalensis*), common palm civet (*Paradoxurus hermaphroditus*), large Indian civet (*Viverra zibetha*), and small Indian civet (*Viverricula indica*). By integrating occupancy and temporal activity analyses, this study advances our understanding of how small carnivores coexist with humans beyond protected areas. We hypothesized that small carnivores persist near human settlements by reducing direct interactions with humans through temporal partitioning and thereby exhibiting activity patterns that differ from those of humans.

## 2. Materials and methods

### 2.1 Study area

We conducted the study during January–February 2023 in the BRC, Madhesh Province, Nepal, a 2,261 km^2^ area comprising forest and agricultural landscapes between the Ratu River in the east and the Bagmati River in the west (Fig 1). The BRC contains the Chure hills in the north and flat plains in the south, with elevations ranging from 60–800 m above sea level [44]. Annual rainfall in the complex is 1,733 mm, and the annual average temperature is 23.6^0^C [45]. The BRC contains private, community managed, and national forests, with a part of the national forest managed by the Sagarnath Forest Development Project.

**Fig 1:**
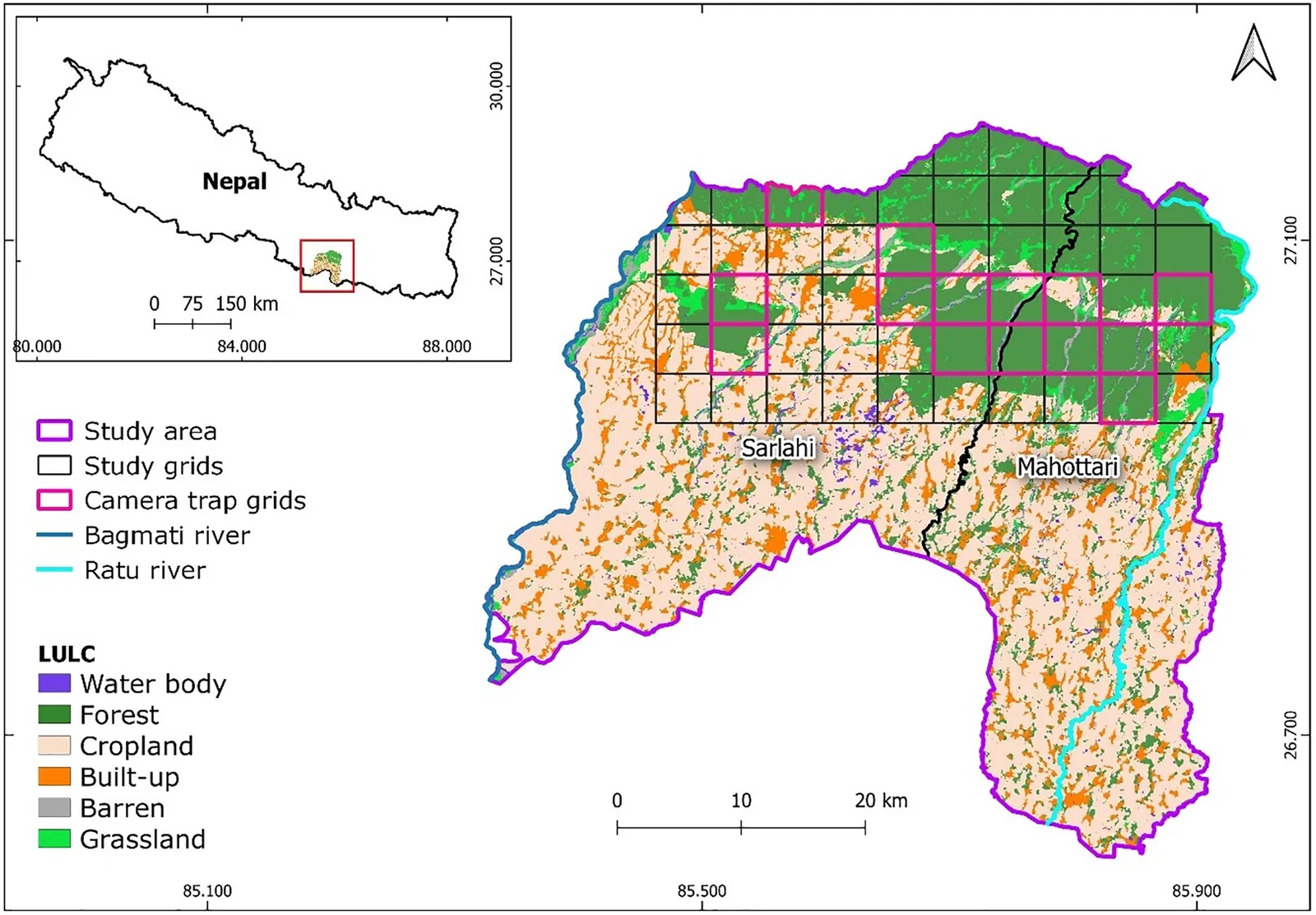
Study area for study of small carnivore occupancy in the Bagmati-Ratu Complex, Nepal, January–February 2023. Contains modified Copernicus Sentinel-2 data (2021) and Esri Land Use and Land Cover (LULC) data, which is made available under a Creative Commons Attribution International License (CC BY 4.0) [46,47].

Major woody vegetation in the BRC includes laurel (*Terminalia alata*), crape myrtle (*Lagerstroemia parviflora*), kamala (*Mallotus philippensis*) and sal (*Shorea robusta*) [33,44]. Mammal species include striped hyena (*Hyaena hyaena*), Bengal fox (*Vulpes bengalensis*), golden jackal (*Canis aureus*), large Indian civet (*Viverra zibetha*), jungle cat (*Felis chaus*), leopard (*Panthera pardus*), northern red muntjac (*Muntiacus vaginalis*), wild boar (*Sus scrofa*), sloth bear (*Melursus ursinus*) and Asian elephant (*Elephas maximus*) [48–51]. Most local communities in the BRC belong to the Madhesi ethnic group, predominantly the Yadav community, who rely on agriculture and livestock farming as their primary livelihood [44,45].

### 2.2 Data collection

We deployed remote cameras (Stealth Cam model STC-G45NG) from 6 January to 20 February 2023 to collect presence/absence data of small carnivores. A total of 34 plots of 5 km x 5 km were established within the forest area of the BRC for camera trapping and the minimum required sample size (n = 20) was selected based on a 95% confidence level and a15% margin of error [52]. We excluded grid plots where farmland and human settlements covered more than 50% of the area to minimize the risk of camera theft and vandalism. The Chure region (> 300m) of the landscape was also avoided due to the challenges of regular monitoring of cameras.

In total 42 cameras were deployed across 14 grids, keeping 3–4 cameras per grid (Fig 1). During the survey, six cameras were damaged due to theft and vandalism; however, data from remaining surveyed sites were used in the analyses. A minimum distance of 1 km was maintained between the cameras and after placing them for 14 days they were subsequently relocated to other sites. Thus, the overall survey period represents the cumulative duration of camera deployment across all sites rather than the sampling duration at a single site. Cameras were installed 40–60 cm above ground along wildlife trails without bait. We programmed cameras to operate continuously throughout the 24-hour diel period, recording 3 images per detection with a 30-second delay between detections.

At each camera trapping station, we measured variables like area of forest, water bodies, human settlements and number of human and livestock detected. We downloaded the ESRI 10-m Annual Land Use Land Cover (LULC) map of 2021, derived from European Space Agency (ESA) Sentinel-2 imagery [46] and the corresponding area of forest, water resource and human settlement within 1 km buffer of the camera station was clipped using QGIS [53]. The land cover area was used to calculate habitat evenness metric using package “landscapemetrics” [54]. The frequency of human and livestock detections needed for the analyses were taken from the photographs of the corresponding camera-trap. The photographs captured in the camera-trap were identified with the help of Indian Mammals: A Field Guide [55].

### 2.3 Ethical considerations and research permits

Camera traps research permission was obtained from the Department of Forest and Soil Conservation (Permission Number: 476). Additionally, we collaborated with and informed the local residents about the purpose of the camera trap survey, advising them not to disturb or damage the equipment.

### 2.4 Inclusivity in global research

Additional information regarding the ethical, cultural, and scientific considerations specific to inclusivity in global research is included in the Supporting Information (S1 Checklist).

### 2.5 Data analysis

The collected data were filtered, arranged, and summarized using the tidyverse package [56] in R Program [57]. A binary detection history was then created for each site, where a numerical value of 1 indicated at least one detection of species *k* at site *i*, while 0 indicated non-detection [23]. The data of seven consecutive camera trap days was pooled into one sampling occasion and altogether two sampling occasions were created for the analysis [58,59]. These repeated sampling occasions allowed detection probability to be estimated separately from occupancy, thereby accounting for imperfect detection in the occupancy model. Although 10 small carnivores were detected during the survey, species with at least five detections were only included in the analysis to prevent model non-convergence and ensure the reliability of the conclusions drawn [58]. However, the number of independent detections was low for some species: leopard cat and small Indian civet each has only 13 independent detections, and small Indian civet was detected at only six stations. These sample sizes limit the precision of species-specific occupancy and habitat-association estimates. However, hierarchical Bayesian multi-species occupancy model borrows strength across species through community-level hyperpriors, which stabilizes species-level estimates. Overall, the two sampling occasions and available detection histories (p > 0.2) were adequate to derive community-level inference on occupancy [23].

Before analysis, all independent variables were assessed for multicollinearity using Pearson’s correlation test. An exclusion threshold of |r| ≥ 0.7 was applied to identify and remove highly correlated variables. However, no pair of variables exceeded this threshold; therefore, all variables were retained for subsequent analyses (S1 Fig). All the continuous variables were z-standardized (mean = 0, standard deviation = 1) prior to analysis [60].

The hierarchical analysis was performed using model developed by Royle & Dorazio [61] and adapted by Regmi et al. [16] to calculate detection probability and occupancy of small carnivores.

The detection probability and occupancy for each species *k* at each site *i* was calculated using the following formula:

For detection probability;


$$\begin{array}{c} ogit(p_{ik})={\alpha \text{0}}_{k}+{\alpha DNR}_{k}+{\alpha DNW}_{k}+{\alpha DHS}_{k} \end{array}$$

For occupancy probability;


$$\begin{array}{c} ogit\;(\psi_{ik})={\beta \text{0}}_{k}+{\beta CC}_{k}+{\beta Farmland}_{k}+{\beta Settlement}_{k}+{\beta NHD}_{k}+{\beta NLD}_{k}+{\beta shdi}_{k} \end{array}$$

where the coefficients (*α* and *β*) differ between species.

We included the correlation between *ψ* and *ρ*, where the intercept *β*_*0,k*_ is the probability of occupancy of species *k* at site *i* with a given combination of covariates and *α*_*0,k*_ is the detection probability of the species. In the given formula DNR = distance to nearest road, DNW = distance to nearest water body, DHS = distance to nearest human settlement, CC = canopy cover percentage, Farmland = area of farmland, Settlement = area of settlement, NHD = numbers of humans detected, NLD = number of livestock detected, and shdi = Shannon’s index of habitat heterogeneity. We used adaptive MCMC with 30,000 iterations, five chains, 3,000 adaptations, and 3,000 burnins to estimate species occupancy. We performed occupancy analyses using Just Another Gibbs Sampler [62] using packages coda [63] and jagsUI [64] in R Program [65]. In JAGS, we specified dnorm(μ_θ, τ_θ) for each species-level regression coefficient and dnorm (0, 0.1) for the community-level mean hyperprior. Similarly, we set dunif (0, 1.5) for the community-level standard deviation hyperprior. The precision was then computed as $\tau_{\theta }=\text{1}/\sigma_{\theta }^{\text{2}}$. Thus, all species-level regression coefficients were drawn from normal distributions whose means and standard deviations were themselves estimated from community-level hyperpriors. Here, θ denotes each of the occupancy and detection coefficients.

The date and time of small carnivore detections were obtained from the timestamp of camera-trap images, and used to create the detection history in an excel sheet. Similarly, the detection time of each species was used to determine and analyze their diel activity patterns. We defined activity pattern of each species as day-time for photographs captured between 0600 and 1759 hours and night-time for those taken during 1800 and 0559 hours [66]. These patterns were further refined by classifying species activities into specific categories: strongly nocturnal (≥85% of observations between 1800–0559 hours), mostly nocturnal (61–84% of observations between 1800–0559 hours), cathemeral (40–60% of activity during day or night), mostly diurnal (61–84% of observations between 0600–1759 hours), or strongly diurnal (≥85% of observations between 0600–1759 hours) [42].

The temporal co-occurrence between the small carnivores was estimated on the basis of their daily activity patterns. We used the exact time recorded on camera trap images to calculate activity overlap coefficients $(\hat{\Delta })$ for the five small carnivores using the overlap package in R program [67]. Based on our independent detection sample sizes, we utilized the ${\hat{\Delta }}_{\text{1}}$ estimator for the coefficient of overlap, as it is recommended for small sample sizes **(**< 50) [42]. The coefficient value ranges from 0 (no overlap) to 1 (complete overlap) and is defined as the area under the curve which is formed by taking minimum of the two density function at each point of time [68]. We considered ${\hat{\Delta }}_{\text{1}}$ > 0.8 to be a strong overlap and 0.5 <${\hat{\Delta }}_{\text{1}}$ < 0.8 as a moderate overlap [42]. A total of 999 bootstraps were employed to generate 95% confidence intervals, and the overlapPlot function was used to plot the calculated overlap [69].

## 3. Results

Overall, we obtained 903 independent images of wild animals (see representative camera-trap photographs of the five small carnivores in Fig 2). Among the small carnivores, jungle cat was detected 23 times at 12 sites, followed by large Indian civet (24 times at 10 sites), common palm civet (16 times in 11 sites), leopard cat (13 times at 9 sites), and small Indian civet (13 times at 6 sites) (S3 Table, Sheet 1). The mean community level detection probability of these five species was 0.273 ± 0.054 and the mean community occupancy was 0.665 ± 0.106.

**Fig 2:**
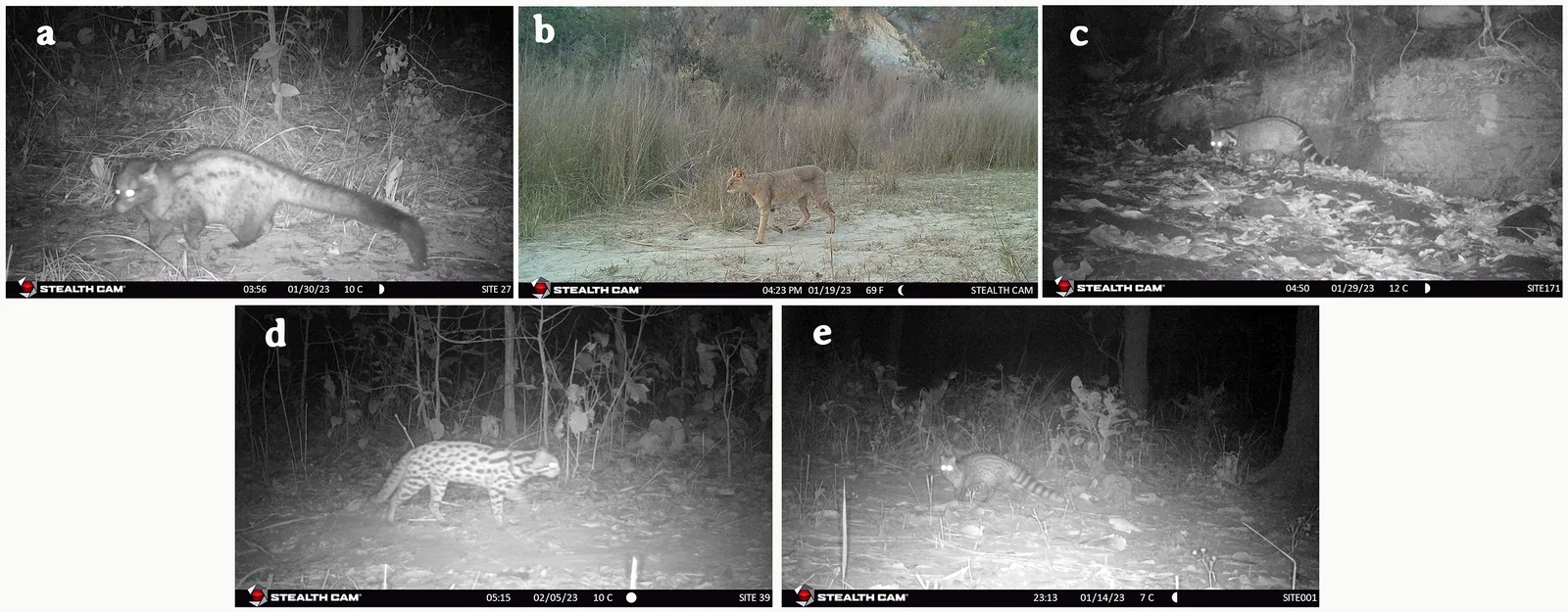
Representative camera-trap photographs of the five small carnivores recorded during the study: (a) common palm civet, (b) jungle cat, (c) large Indian civet, (d) leopard cat, and (e) small Indian civet.

Detection probability of small carnivore species ranged from 0.245 to 0.315, with the greatest detection probability for jungle cat (*p* = 0.315 ± 0.078) followed by large Indian civet (*p* = 0.289 ± 0.075) and lowest for leopard cat (*p* = 0.245 ± 0.063) (Fig 3 and S1 Table). Similarly, the detection probability for common palm civet was 0.248 ± 0.067 and for small Indian civet was 0.270 ± 0.066. Occupancy was higher for common palm civet (*ψ* = 0.698 ± 0.134) followed by jungle cat (*ψ* = 0.669 ± 0.129). Similarly, the occupancy for small Indian civet was 0.655 ± 0.124 and 0.652 ± 0.115 for leopard cat. The lowest occupancy was for large Indian civet (*ψ* = 0.651 ± 0.123) (Figs 3 and 4 and S1 Table).

**Fig 3:**
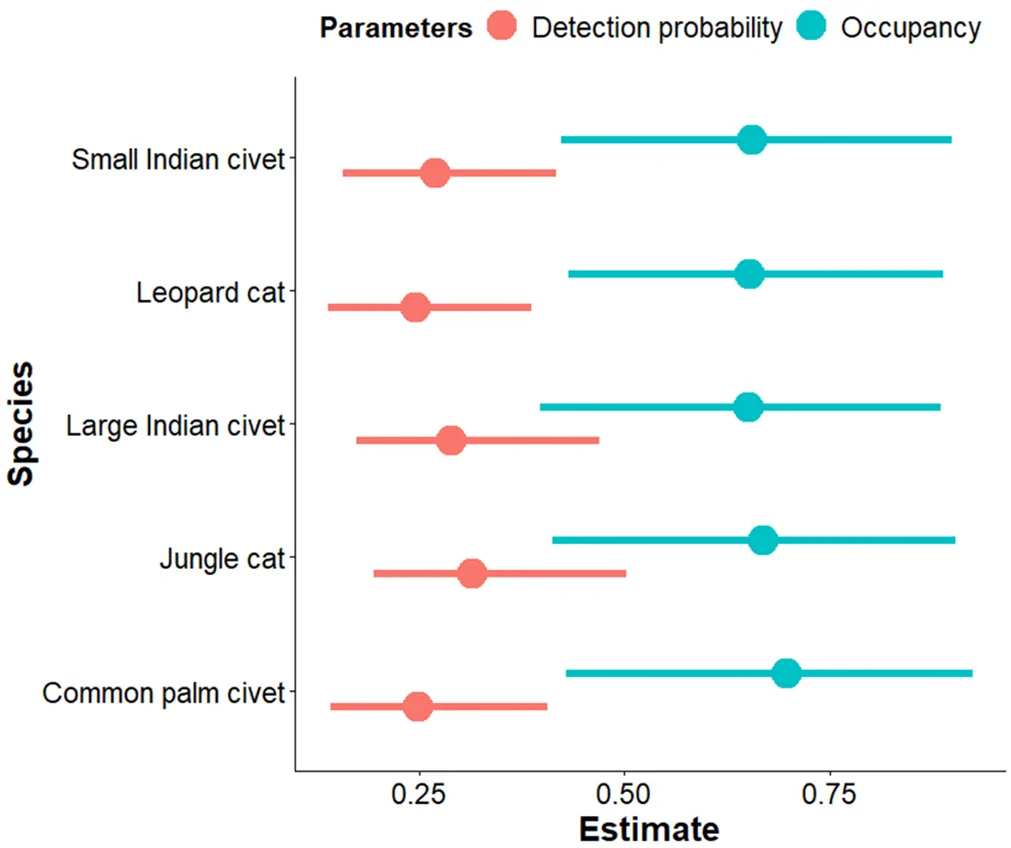
Species level detection probability (p) and occupancy (ψ) for five small carnivores in Bagmati-Ratu Complex, Nepal, January–February 2023.

**Fig 4:**
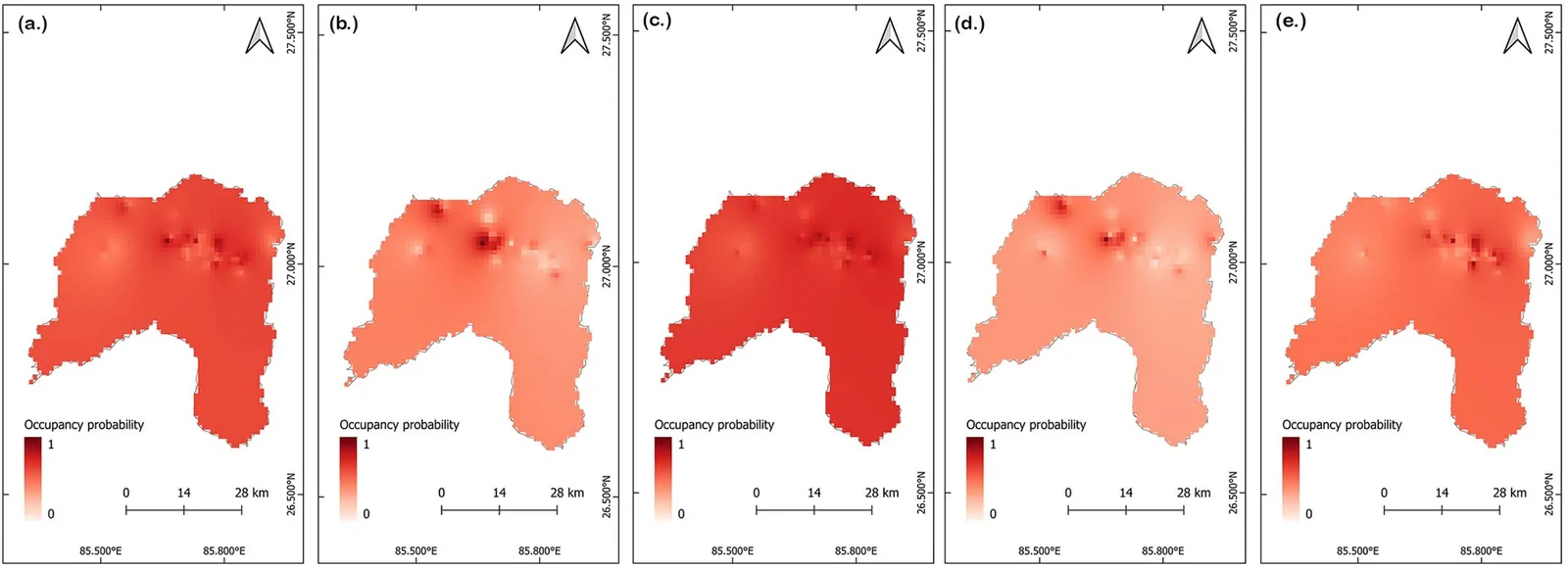
Inverse Distance Weighting (IDW) interpolation-based spatial occupancy probability maps of five small carnivores detected in the Bagmati-Ratu Complex, Nepal: (a) common palm civet, (b) jungle cat, (c) leopard cat, (d) large Indian civet, and (e) small Indian civet.

The mean canopy cover across the study area was 56.125 ± 26.030%, mean area of farmland was 0.034 ± 0.084km^2^, and settlement was 0.010 ± 0.043 km^2^. The average number of times human and livestock detected was 103.1 ± 76.0 and 71.8 ± 96.8, respectively. The mean habitat evenness in the area was 0.237 ± 0.241. The average distance to road from the camera trap location was 425.508 ± 332.734m, distance to settlement was 1,297.069 ± 728.593m, and distance to water was 1,069.306 ± 847.969m. Small carnivore community occupancy decreased with increasing area of farmland (−2.200 ± 1.168), and the Bayesian credible interval did not overlap zero (Table 1). However, there was no association between other covariates and community level occupancy of small carnivores.

**Table 1:** Community level summaries of covariates for occupancy and detection probabilities of five small carnivores in Bagmati-Ratu Complex, Nepal, January–February 2023. Mean (Mu), standard deviation (SD), lower confidence interval (LCI), median (Md), upper confidence interval (UCI), Rhat, effective sample size (ESS), overlap0 (proportion of posterior with same size), and f statistics (F; the posterior probability of an effect) of the distribution.

Parameters	Mean	SD	LCI	UCI	Rhat	ESS	overlap0	F statistics
**Occupancy model**								
**Intercept**	2.443	1.662	−0.121	6.094	1.017	226	1	0.964
**Canopy cover (%)**	−1.536	1.190	−4.089	0.513	1.013	317	1	0.926
**Number of humans detected**	0.369	1.277	−1.781	3.435	1.003	1566	1	0.576
**Number of livestock detected**	1.649	1.415	−0.642	4.770	1.016	207	1	0.899
**Settlement area**	2.630	1.894	−0.050	7.092	1.003	1683	1	0.971
**Habitat evenness**	0.436	0.764	−1.040	2.021	1.005	923	1	0.733
**Farmland area**	**−2.200**	**1.168**	**−4.722**	**−0.290**	**1.016**	**211**	**0**	**0.988**
**Detection model**								
**Intercept**	−1.142	0.361	−1.786	−0.344	1.006	512	0	0.995
**Distance to road**	0.170	2.278	−4.426	4.349	1.007	1398	1	0.548
**Distance to water body**	0.206	0.379	−0.562	0.985	1.000	31486	1	0.732
**Distance to settlement**	−0.700	2.276	−4.872	3.884	1.007	1420	1	0.635

Area of farmland was the most important variable for predicting small carnivore occupancy across most species (Fig 5 and S2 Table). The occupancy of each small carnivore decreased with increasing area of farmland, with the strongest effect on leopard cats (coefficient = −2.398 ± 1.304) and the weakest on common palm civets (coefficient = −2.121 ± 1.225; S2 Table). However, the effect of farmland area on small Indian civets was not significant. Although not significant at the community level, area of settlement had significant positive association with occupancy of individual small carnivore species, with the strongest association for jungle cats (coefficient = 2.872 ± 1.953) and large Indian civets (coefficient = 2.739 ± 1.976) and the weakest for common palm civets (coefficient = 2.673 ± 1.939). However, the effect was not significant for leopard cats and small Indian civets. These inferences should be interpreted with caution because grids dominated (>50%) by farmland and settlements were excluded from sampling to reduce the risk of camera theft.

**Fig 5:**
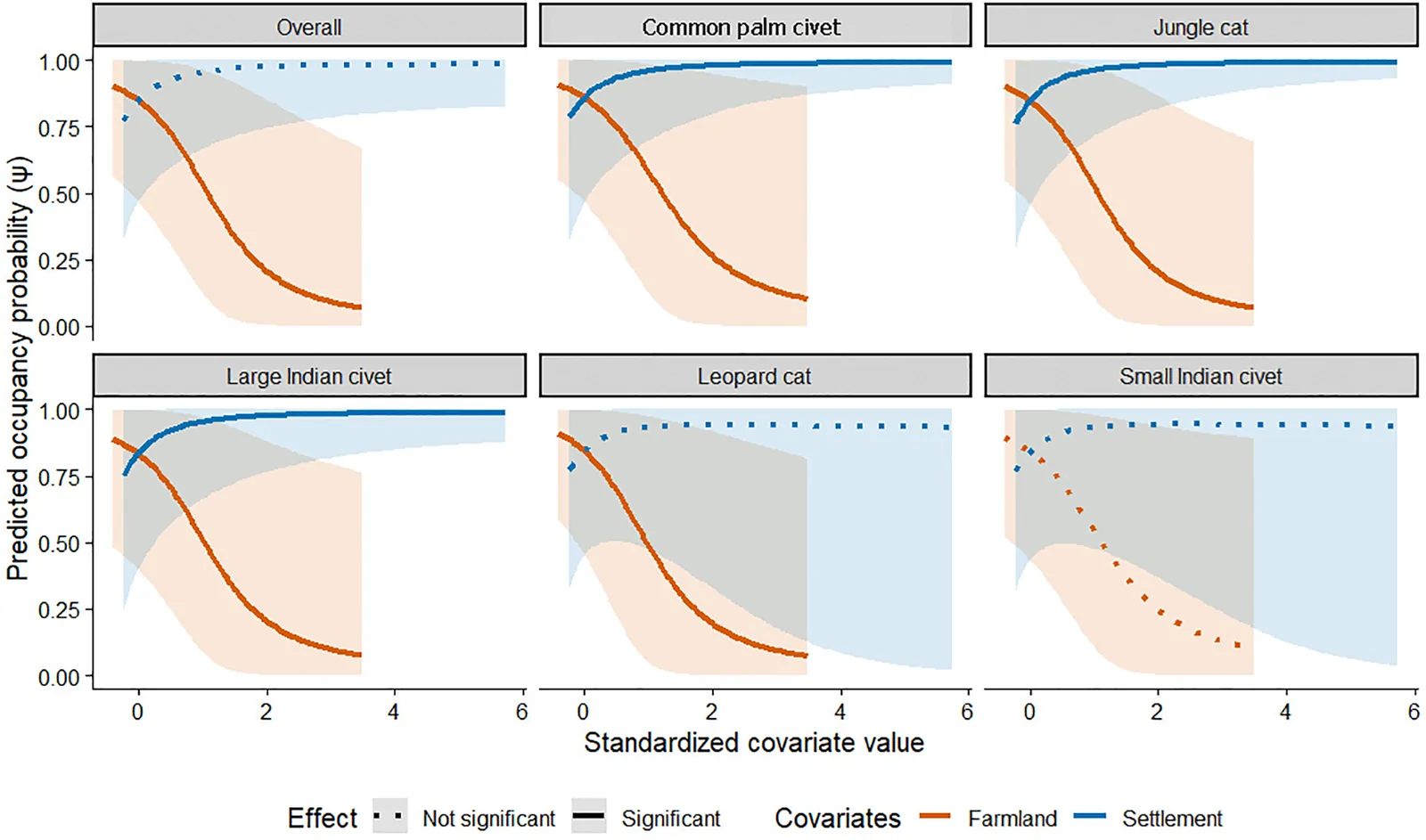
Effects of covariates on occupancy of five small carnivores in Bagmati-Ratu Complex, Nepal, January–February 2023.

We found consistent activity patterns across the five species, with all being strongly nocturnal (91%, n = 81), except the jungle cat, which exhibited some diurnal activity (22%, n = 5) (Table 2). Furthermore, the leopard cat and the small Indian civet were not detected even a single time during the day, whereas jungle cat had the highest number of daytime detections (Tables 2 and S3). None of the species exhibited cathemeral activity patterns.

**Table 2:** Summary of camera-trap encounters, spatial distribution, and diel activity patterns for five small carnivores encountered in Bagmati-Ratu Complex, Nepal.

			No. of observations			Observation (%)		
S.No	Species	Stations observed	Day	Night	Total	Day	Night	Refined category
1.	Jungle cat	12	5	18	23	22	78	Mostly nocturnal
2.	Leopard cat	9	0	13	13	0	100	Strongly nocturnal
3.	Common palm civet	11	1	15	16	6	94	Strongly nocturnal
4.	Large Indian civet	10	2	22	24	8	92	Strongly nocturnal
5.	Small Indian civet	6	0	13	13	0	100	Strongly nocturnal

All species pairs exhibited moderate overlap (0.5 <${\hat{\Delta }}_{\text{1}}$ < 0.8), and none showed a strong overlap in their activity patterns (Fig 6 and S4 Table). The highest degree of daily activity overlap was between common palm civet and small Indian civet (Dhat1 = 0.77, CI = 0.67–1.07) followed by leopard cat and jungle cat (Dhat1 = 0.74, CI = 0.61–0.99). In contrast, the large Indian civet and leopard cat exhibited the lowest activity overlap (Dhat1 = 0.58, CI = 0.39–0.8).

**Fig 6:**
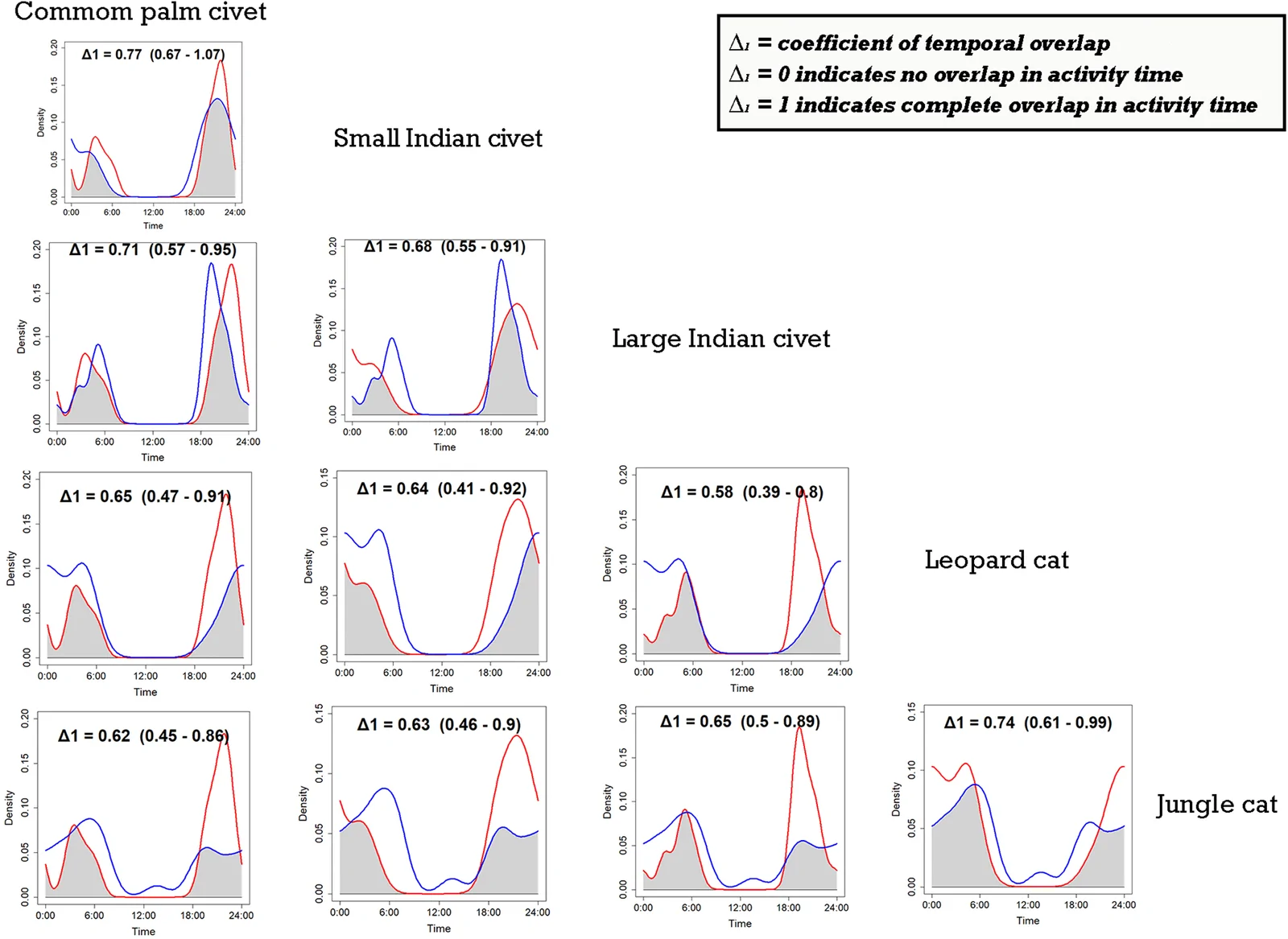
Overlap of activity pattern between small carnivores in Bagmati-Ratu Complex, Madhesh Province, Nepal, January–February 2023. Red lines represent species in columns, blue line represents species in rows, and temporal overlap is represented by gray shaded areas. Number indicates coefficient of overlap, ${\hat{\Delta }}_{\text{1}}$ and confidence interval is listed in parenthesis.

## 4. Discussion

The camera-trap study provides valuable information on occupancy, detection probability and activity patterns of five small carnivores in a human-dominated landscape of Madhesh Province, Nepal. The relatively higher detection probabilities for large Indian civet and jungle cat and least for leopard cat and common palm civet were more informative findings from this study. The higher detection probability of large Indian civet might be due to its widespread distribution in the area [70] or its larger home range size among other small carnivores [43,71]. Jungle cats being active during both daytime as well as nighttime may have contributed to their highest detection probability [20].

We observed the highest occupancy estimates for common palm civets and jungle cats. Common palm civets can use both ground-level as well as upper tree canopy as habitat [72] which might be the reason for their higher occupancy across study sites. The higher occupancy and frequent detection of jungle cat in the study area might be associated with its preference for domestic prey [34], as the study was conducted in a human-dominated landscape. Previous studies have reported that the occupancy of jungle cat outside protected area to be higher in areas with lower canopy cover [19] which occur near human settlements and our study area is one of the densely human populated area in Madhesh Province of Nepal [73]. Despite having higher detection probability, the occupancy of large Indian civet was the lowest in the study area, potentially due to its preference towards dense forest areas that provide adequate cover [74], but the species may also occur near human settlements connected with large forest patches [75]. Additionally, high levels of human disturbance in the BRC may have reduced the potential distribution of large Indian civet [8]. However, these explanations require further investigation to verify it.

We observed a significant negative association between area of farmland and community-level occupancy of small carnivores. The negative association with farmland may be related to intensive agricultural practices in the Tarai, including monoculture and pesticide use, which may reduce the availability of prey like rodents and lagomorphs. Similarly, the structural homogeneity of farmland may provide limited refuge from predation or persecution compared to areas with complex vegetation mosaics [76,77]. However, because our analysis was observational, this association does not establish a causal relationship between farmland extent and small carnivore occupancy. Farmland area may also be correlated with other landscape characteristics, including vegetation structure, human activity, prey availability, and habitat fragmentation, which might contribute to the observed pattern. The non-significant association of settlement area at the community level suggests that the positive resource subsidies previously reported in other systems may be offset by mortality risk in the study area due to feral dog predation and vehicular collisions [78–80], potentially resulting in a net neutral effect at the occupancy level. Further, it may also be possible that small carnivores may use peripheries of settlements while avoiding core areas [8,81], a fine-scale behavioral response that may occur but our grid-cell occupancy may not have captured. These findings align with studies that show constrained mesopredator distribution in intensively modified landscapes [78–82]. The predominant negative association between farmland area and small carnivore occupancy suggests that agricultural expansion may pose the primary landscape-level threat to small carnivores, potentially escalating conflicts in fragmented agricultural matrices [83,84].

At the species level, however, we observed a significant positive association of jungle cats, large Indian civets, and common palm civets with settlement area. This highlights that, rather than community-level patterns, species-specific ecological traits may contribute to differences in responses to human-dominated landscapes [85,86]. The positive association may be related to the generalist behavior and ecological plasticity of these species. As highly adaptive mesopredators, they could exploit resources associated with human-modified habitats, including food resources, prey, or shelter around settlements. However, increased occupancy or detection around settlements could also reflect animal movement patterns or camera placement rather than a true preference for human-modified habitats. Therefore, these positive associations should be interpreted cautiously and do not necessarily indicate attraction to or preference for settlements. The contrasting responses between community-level negative association with farmland area and species-specific positive associations with settlement area highlight the need to consider species-specific ecology in conservation planning. While agricultural expansion poses a primary threat to the overall small carnivore guild, certain generalist species may exhibit dietary and behavioral flexibility to persist and benefit from human modifications [85,87]. Given the negative association between farmland area and small carnivore occupancy, maintaining natural or semi-natural habitat elements within agricultural landscapes may help retain suitable habitat for small carnivores. Such habitats may include forest patches and vegetated areas that can provide cover and resources within agricultural landscapes. This differential response highlights the need for targeted management strategies that account for species-specific vulnerabilities and adaptations within human-dominated landscapes of the Tarai.

Small carnivores did not exhibit much difference in their diel activity patterns, indicating temporal co-occurrence rather than avoidance. All species exhibited nocturnal behavior and ceased activity during daytime, potentially to avoid human interaction. Small carnivores may alter their activity in human-dominated landscapes to avoid temporal overlap with humans [88]. Jungle cats exhibited bimodal activity, showing some movement during the day, though peak activity occurred during the early morning and early evening, as reported in the Parsa-Koshi Complex and Bardiya in Nepal [19,41] as well as in Srepok Wildlife Sanctuary of Cambodia [39]. However, given the small number of daytime observations for jungle cats (n = 5), this pattern and the distinction between mostly and strongly nocturnal activity should be interpreted cautiously. Past research describes the activity patterns of jungle cats variably, ranging from bimodal [89,90] to strongly nocturnal [19]. Previous studies across different regions of Asia have consistently reported leopard cats to be strongly nocturnal [19,39,82,91], which corroborates our findings, as almost all detections occurred at night. In the present study, common palm civet and small Indian civet exhibited high temporal overlap, likely reflecting their predominantly nocturnal habits. Such temporal overlap may be facilitated by partitioning along other niche dimensions, including diet and microhabitat use, which may reduce direct competition between these species, as reported in Myanmar [72]. Both species exploit a variety of resources, including rodents, insects, and fruits, and are known to tolerate human-modified environments [92,93]. A relatively lower activity overlap was observed between large Indian civet and leopard cat. Large Indian civets exhibited peak activity during dusk and dawn rather than throughout the night and generally prefer closed-canopy forests [21,74], whereas leopard cats were more nocturnal, exhibiting peak activity during nighttime, a pattern also reported from Bardiya National Park [41] and the Parsa-Koshi Complex of Nepal [19], and are considered habitat generalists [70]. Such differences in activity patterns and habitat use may contribute to temporal segregation and reduce direct competition between the species. However, camera-trap detections may also be influenced by habitat characteristics, camera placement, animal movement, and species detectability, which should be considered when interpreting the observed diel activity patterns.

Our study has some limitations, such that cameras were deployed only during winter and could not be replicated during the same season in consecutive years. Additionally, due to the challenges in accessing the Chure Hills, sampling was restricted to lower elevations (<300 m) of the BRC, and camera traps could not be deployed in grids with > 50% of their area occupied by farmland and settlements because of the high risk of camera theft and vandalism. Moreover, six cameras were damaged due to theft and vandalism during the survey, and data from these sampling sites could not be incorporated into the analyses, thereby further reducing the spatial coverage of the survey. Therefore, the single-season design and limited spatial and temporal coverage warrant caution when extrapolating these findings across years and unsampled areas of the BRC. In particular, the occupancy and activity patterns reported here represent estimates for the study period and may not necessarily reflect seasonal or annual patterns, as variation in habitat use, prey availability, agricultural activities, and human disturbance could influence occupancy and diel activity patterns [82]. In addition, the relatively low number of independent detections for leopard cat and small Indian civet may constrain species-specific inference. Although the hierarchical model borrows strength across species, their occupancy and covariate estimates should be considered provisional and interpreted with caution. Nevertheless, this research is among the limited studies of small carnivores conducted in this region and addresses knowledge gap of small carnivores outside protected areas in Nepal. Thus, we believe our study provides valuable baseline information on the occurrence and habitat preference of small carnivores in the sampled lowland and human-dominated landscapes of the BRC and will be useful for informing conservation and coexistence practices. This study suggests future research that encompasses broader temporal and spatial scales, including higher elevations and a wide range of human-dominated habitats, to better understand the occupancy and habitat use of small carnivores in the region.

## 5. Conclusions

Our study highlighted the relationships between small carnivores and humans across the surveyed landscape of the BRC. The observed positive associations between small carnivore occupancy and human settlement suggest that some species occurred in areas with more settlement within the sampled landscape. However, these associations should not be interpreted as evidence of species-wide adaptation to human-modified environments, particularly in areas with high human density that were not represented in our sampling. Small carnivores altered their activity to avoid human interactions, being strongly nocturnal. Common palm civet and small Indian civet exhibited highest temporal overlap, as they share similar ecological characteristics and often respond similarly to environmental conditions. The study highlights the importance of considering urban and other human-dominated areas beyond protected areas in small carnivore conservation planning. However, given the limited sampling of human-dominated habitats, our results should be regarded as preliminary and interpreted with caution with respect to human–small carnivore interactions. Further studies encompassing broader spatial and temporal scales are required to better understand human–small carnivore relationships and to develop effective coexistence strategies in the BRC and other human-dominated landscapes.

## Supporting information

S1 Fig Pearson correlation matrix of nine predictor variables to estimate factors affecting occupancy of five small carnivores in Bagmati-Ratu River Complex, Madhesh Province, Nepal. (TIFF)

S2 Fig Variance inflation factor (VIF) analysis of the nine environmental and anthropogenic predictor variables included in the occupancy models. (TIFF)

S1 Table Occupancy and detection probability of five small carnivores in Bagmati Ratu Complex, Nepal, January–February 2023. Mean (Mu), standard deviation (SD), lower confidence interval (LCI), median (Md), upper confidence interval (UCI), Rhat, effective sample size (ESS), overlap0 (proportion of posterior with same size), and f statistics (F) of the distribution.
(DOCX)

S2 Table Effect of variables on species level occupancy of five small carnivores in Bagmati Ratu Complex, Nepal, January–February 2023. (DOCX)

S3 Table Camera-trap records of five small carnivore species in the Bagmati-Ratu Complex, Nepal. Sheet 1 summarizes species detections, including the number of detections by species and diel period (day and night). Sheet 2 contains the raw camera-trap detection records, including camera-trap station ID, date and time of capture, and species identity for each independent detection event.
(XLSX)

S4 TableEstimates of activity pattern overlap ${(\hat{\Delta }}_{1})$ among five small carnivore species in the Bagmati-Ratu Complex, Nepal, from January to February 2023.Perfect overlap between species pairs is represented by 1 (identical activity), with approximate 95% bootstrap confidence intervals indicated in parentheses. CPC = Common palm civet, SIC = Small Indian civet, LIC = Large Indian civet, LC = Leopard cat, JC = Jungle cat.(DOCX)

S1 Checklist PLOS’ questionnaire on inclusivity in global research. (DOCX)
