# Supplementary material for: Spatio-temporal occurrence of small carnivores in Bagmati-Ratu Complex, Nepal
Source: PLoS One. 2026 Nov 13;21(11):e0362681. doi: 10.1371/journal.pone.0362681 (PMC13766245; doi:10.1371/journal.pone.0362681)
Supplement: Supplementary file 1 [file pone.0362681.s003.docx]

**S1 Table. Occupancy and detection probability of five small carnivores in Bagmati Ratu Complex, Nepal, January–February 2023.** Mean (Mu), standard deviation (SD), lower confidence interval (LCI), median (Md), upper confidence interval (UCI), Rhat, effective sample size (ESS), overlap0 (proportion of posterior with same size), and f statistics (F) of the distribution.

| **Species** | **parameters** | **Mu** | **SD** | **LCI** | **Md** | **UCI** | **Rhat** | **ESS** | **Overlap0** | **F** |
| --- | --- | --- | --- | --- | --- | --- | --- | --- | --- | --- |
| **Common palm civet** | psi | 0.698 | 0.134 | 0.43 | 0.69 | 0.91 | 1 | 1021 | 0 | 1 |
|  | p | 0.245 | 0.066 | 0.14 | 0.25 | 0.40 | 1 | 1102 | 0 | 1 |
| **Jungle cat** | psi | 0.669 | 0.129 | 0.41 | 0.66 | 0.89 | 1 | 1059 | 0 | 1 |
|  | p | 0.315 | 0.078 | 0.20 | 0.31 | 0.50 | 1 | 5067 | 0 | 1 |
| **Large Indian civet** | psi | 0.651 | 0.123 | 0.39 | 0.64 | 0.87 | 1 | 1142 | 0 | 1 |
|  | p | 0.289 | 0.075 | 0.18 | 0.29 | 0.47 | 1 | 5185 | 0 | 1 |
| **Leopard cat** | psi | 0.652 | 0.115 | 0.44 | 0.64 | 0.87 | 1 | 4757 | 0 | 1 |
|  | p | 0.248 | 0.067 | 0.13 | 0.25 | 0.38 | 1 | 1845 | 0 | 1 |
| **Small Indian civet** | psi | 0.655 | 0.124 | 0.44 | 0.65 | 0.88 | 1 | 4073 | 0 | 1 |
|  | p | 0.270 | 0.066 | 0.16 | 0.27 | 0.41 | 1 | 4436 | 0 | 1 |
