# Supplementary material for: Spatio-temporal occurrence of small carnivores in Bagmati-Ratu Complex, Nepal
Source: PLoS One. 2026 Nov 13;21(11):e0362681. doi: 10.1371/journal.pone.0362681 (PMC13766245; doi:10.1371/journal.pone.0362681)
Supplement: Supplementary file 2 [file pone.0362681.s004.docx]

**S2 Table. Effect of variables on species level occupancy of five small carnivores in Bagmati Ratu Complex, Nepal, January–February 2023.**

| **Species** | **Parameters** | **Mean** | **SD** | **LCI** | **Rhat** | **ESS** | **overlap0** | **f** |
| --- | --- | --- | --- | --- | --- | --- | --- | --- |
| Common palm civet | **Occupancy** | | | | | | | |
|  | Intercept | 2.679 | 1.839 | -0.127 | 1.015 | 247 | 1 | 0.964 |
|  | Canopy cover (%) | -0.484 | 1.741 | -3.850 | 1.008 | 401 | 1 | 0.594 |
|  | Number of humans detected | 0.225 | 1.501 | -2.524 | 1.002 | 2369 | 1 | 0.524 |
|  | Number of livestock detected | 2.117 | 1.392 | -0.164 | 1.012 | 263 | 1 | 0.963 |
|  | Settlement area | 2.673 | 1.939 | 0.017 | 1.002 | 1893 | 0 | 0.976 |
|  | Farmland area | -2.121 | 1.225 | -4.745 | 1.015 | 221 | 0 | 0.977 |
|  | Habitat evenness | 0.539 | 0.828 | -1.002 | 1.003 | 2441 | 1 | 0.761 |
|  | **Detection probability** | | | | | | | |
|  | Intercept | -1.242 | 0.387 | -1.980 | 1.005 | 599 | 0 | 0.996 |
|  | Distance to road | 0.405 | 2.314 | -4.259 | 1.007 | 1297 | 1 | 0.586 |
|  | Distance to water body | -0.344 | 0.460 | -1.270 | 1.001 | 4508 | 1 | 0.768 |
|  | Distance to settlement | -0.471 | 2.315 | -4.738 | 1.006 | 1303 | 1 | 0.596 |
| Jungle cat | **Occupancy** | | | | | | | |
|  | Intercept | 2.499 | 1.763 | -0.170 | 1.015 | 251 | 1 | 0.959 |
|  | Canopy cover (%) | -1.790 | 1.144 | -4.246 | 1.010 | 469 | 1 | 0.964 |
|  | Number of humans detected | 0.691 | 1.282 | -1.442 | 1.003 | 1184 | 1 | 0.695 |
|  | Number of livestock detected | 1.024 | 1.687 | -1.761 | 1.014 | 240 | 1 | 0.690 |
|  | Settlement area | 2.872 | 1.953 | 0.099 | 1.002 | 1858 | 0 | 0.982 |
|  | Habitat evenness | 0.764 | 0.831 | -0.729 | 1.004 | 1706 | 1 | 0.846 |
|  | Farmland area | -2.287 | 1.279 | -5.091 | 1.014 | 239 | 0 | 0.985 |
|  | **Detection probability** | | | | | | | |
|  | Intercept | -0.979 | 0.421 | -1.655 | 1.006 | 565 | 1 | 0.975 |
|  | Distance to road | -0.033 | 2.318 | -4.721 | 1.007 | 1429 | 1 | 0.488 |
|  | Distance to water body | 0.170 | 0.367 | -0.524 | 1.000 | 11135 | 1 | 0.680 |
|  | Distance to settlement | -0.903 | 2.317 | -5.211 | 1.007 | 1413 | 1 | 0.664 |
| Large Indian civet | **Occupancy** | | | | | | | |
|  | Intercept | 2.385 | 1.768 | -0.411 | 1.015 | 261 | 1 | 0.937 |
|  | Canopy cover (%) | -2.125 | 1.421 | -5.048 | 1.009 | 435 | 1 | 0.949 |
|  | Number of humans detected | 0.127 | 1.524 | -2.653 | 1.002 | 1851 | 1 | 0.495 |
|  | Number of livestock detected | 0.924 | 1.870 | -2.404 | 1.013 | 247 | 1 | 0.660 |
|  | Settlement area | 2.739 | 1.976 | 0.004 | 1.002 | 1710 | 0 | 0.975 |
|  | Habitat evenness | 0.666 | 0.850 | -0.872 | 1.004 | 1581 | 1 | 0.807 |
|  | Farmland area | -2.243 | 1.287 | -5.001 | 1.014 | 235 | 0 | 0.979 |
|  | **Detection probability** | | | | | | | |
|  | Intercept | -1.073 | 0.401 | -1.737 | 1.005 | 659 | 0 | 0.985 |
|  | Distance to road | 0.056 | 2.314 | -4.643 | 1.007 | 1451 | 1 | 0.528 |
|  | Distance to water body | 0.246 | 0.363 | -0.431 | 1.000 | 24460 | 1 | 0.756 |
|  | Distance to settlement | -0.822 | 2.314 | -5.119 | 1.007 | 1401 | 1 | 0.651 |
| Leopard cat | **Occupancy** | | | | | | | |
|  | Intercept | 2.403 | 1.670 | -0.174 | 1.017 | 240 | 1 | 0.960 |
|  | Canopy cover (%) | -1.880 | 1.283 | -4.664 | 1.011 | 373 | 1 | 0.954 |
|  | Number of humans detected | 0.276 | 1.561 | -2.532 | 1.002 | 2660 | 1 | 0.537 |
|  | Number of livestock detected | 1.875 | 1.480 | -0.505 | 1.013 | 248 | 1 | 0.927 |
|  | Settlement area | 2.511 | 2.131 | -0.949 | 1.002 | 2496 | 1 | 0.908 |
|  | Habitat evenness | 0.585 | 0.866 | -1.048 | 1.005 | 874 | 1 | 0.772 |
|  | Farmland area | -2.398 | 1.304 | -5.220 | 1.011 | 296 | 0 | 0.984 |
|  | **Detection probability** | | | | | | | |
|  | Intercept | -1.229 | 0.354 | -1.924 | 1.004 | 790 | 0 | 0.999 |
|  | Distance to road | 0.367 | 2.315 | -4.294 | 1.006 | 1452 | 1 | 0.579 |
|  | Distance to water body | 0.249 | 0.337 | -0.410 | 1.000 | 39062 | 1 | 0.778 |
|  | Distance to settlement | -0.514 | 2.317 | -4.800 | 1.006 | 1490 | 1 | 0.604 |
| Small Indian civet | **Occupancy** | | | | | | | |
|  | Intercept | 2.406 | 1.743 | -0.252 | 1.017 | 219 | 1 | 0.952 |
|  | Canopy cover (%) | -1.537 | 1.232 | -4.194 | 1.011 | 360 | 1 | 0.920 |
|  | Number of humans detected | 0.562 | 1.277 | -1.568 | 1.003 | 1634 | 1 | 0.650 |
|  | Number of livestock detected | 2.455 | 1.448 | 0.096 | 1.013 | 258 | 0 | 0.980 |
|  | Settlement area | 2.541 | 2.129 | -0.874 | 1.002 | 1896 | 1 | 0.912 |
|  | Habitat evenness | -0.350 | 1.287 | -3.085 | 1.006 | 513 | 1 | 0.574 |
|  | Farmland area | -2.069 | 1.237 | -4.748 | 1.015 | 220 | 1 | 0.974 |
|  | **Detection probability** | | | | | | | |
|  | Intercept | -1.212 | 0.372 | -1.928 | 1.005 | 569 | 0 | 0.998 |
|  | Distance to road | 0.059 | 2.313 | -4.603 | 1.006 | 1638 | 1 | 0.527 |
|  | Distance to water body | 0.722 | 0.448 | -0.050 | 1.000 | 9155 | 1 | 0.965 |
|  | Distance to settlement | -0.813 | 2.302 | -5.077 | 1.006 | 1571 | 1 | 0.650 |
