# Supplementary material for: Spatio-temporal occurrence of small carnivores in Bagmati-Ratu Complex, Nepal
Source: PLoS One. 2026 Nov 13;21(11):e0362681. doi: 10.1371/journal.pone.0362681 (PMC13766245; doi:10.1371/journal.pone.0362681)
Supplement: Supplementary file 3 [file pone.0362681.s006.docx]

**S4 Table. Estimates of activity pattern overlap** ${\boldsymbol{(}\hat{\boldsymbol{\Delta}}}_{\boldsymbol{1}}\boldsymbol{)}$ **among five small carnivore species in the Bagmati-Ratu Complex, Nepal, from January to February 2023.** Perfect overlap between species pairs is represented by 1 (identical activity), with approximate 95% bootstrap confidence intervals indicated in parentheses. CPC = Common palm civet, SIC = Small Indian civet, LIC = Large Indian civet, LC = Leopard cat, JC = Jungle cat.

| **CPC** | **SIC** | **LIC** | **LC** | **JC** |  |
| --- | --- | --- | --- | --- | --- |
| 1 |  |  |  |  | **CPC** |
| 0.77  (0.67-1.07) | 1 |  |  |  | **SIC** |
| 0.71  (0.57-0.95) | 0.68  (0.55-0.91) | 1 |  |  | **LIC** |
| 0.65  (0.47-0.91) | 0.64  (0.41-0.92) | 0.58  (0.39-0.8) | 1 |  | **LC** |
| 0.62  (0.45-0.86) | 0.63  (0.46-0.9) | 0.65  (0.5-0.89) | 0.74  (0.61-0.99) | 1 | **JC** |
